# Survey data on consumer responses to social media-endorsed FMCG advertising in Vietnam

**DOI:** 10.1016/j.dib.2026.113060

**Published:** 2026-07-07

**Authors:** Thuy Duong Pham, Huong Thi Lan Le, Hai Ninh Nguyen

**Affiliations:** a National Economics University (NEU), Vietnam; b School of International Business and Economics, Foreign Trade University, Vietnam

**Keywords:** Influencer marketing, Digital consumer behavior, Endorsement, Brand association, Customer Y experience, Online advertising, Survey dataset, Emerging market

## Abstract

This article presents a survey dataset on consumer responses to social media-endorsed advertising in Vietnam’s fast-moving consumer goods (FMCG) sector. Data were collected through a cross-sectional online survey administered in Google Forms and distributed via relevant Facebook groups in Vietnam between August and December 2025, using a non-probability purposive sampling approach targeting adult social media users with recent exposure to influencer-endorsed FMCG advertising. Of the 1003 submitted responses, 563 were retained in the refined analytical dataset after informed consent screening, five eligibility screening questions, and post-screening quality checks. The instrument includes 36 Likert-type indicators measuring nine reflective constructs (credibility, expertise, congruence, authenticity, entertainment, informativeness, customer experience, brand association, and engagement), together with demographic and control variables. Measurement quality was assessed in SmartPLS 4, with results indicating satisfactory reliability, convergent validity, and discriminant validity for the reflective measurement scales. The repository package contains the raw dataset, the refined analytical dataset, the codebook, the informed consent form, and the questionnaire in English and Vietnamese. These data can be reused for scale assessment, replication, multigroup comparison, mediation analysis, and comparative research on influencer-led advertising and brand-related outcomes in emerging markets.

Specifications Table

**Table:** 

Subject	Social Sciences
Specific subject area	Social media marketing, influencer-endorsed advertising, consumer behavior, FMCG branding.
Type of data	Table; raw survey dataset; refined survey dataset; codebook; questionnaire; informed consent form.
Data collection	Structured online questionnaire administered through Google Forms and distributed via relevant Facebook groups in Vietnam between August and December 2025, using non-probability purposive sampling. Respondents provided informed consent and completed five eligibility screening questions. Of the 1003 submitted responses, 563 were retained in the refined analytical dataset after screening and quality checks.
Data source location	Vietnam.
Data accessibility	Repository: Mendeley Data; Dataset title: Survey dataset on social media-endorsed advertising in Vietnam’s FMCG sector Repository name: Mendeley Data Data identification number: (m3n2zwc7tz.1) Direct URL to data: http://doi.org/10.17632/m3n2zwc7tz.1 Instructions for accessing these data: Direct download via the Mendeley Data DOI link above; the repository is openly accessible without registration.
Related research article	No.

## 1 Value of the Data

• These data are useful for examining how consumers evaluate social media-endorsed FMCG advertising through source-related and content-related cues, and how those evaluations relate to customer experience, brand association, and engagement outcomes.
• The dataset is relevant for researchers working on influencer marketing, social media advertising, brand responses, and digital consumer behavior, particularly in emerging-market contexts.
• The data may be reused for descriptive analysis, scale assessment, mediation testing, multigroup comparison, predictive modeling, and cross-context replication.
• The repository package is methodologically useful because it includes both a raw dataset and a refined analytical dataset, allowing researchers to inspect consent screening, eligibility filtering, post-screening data-quality refinement, and variable coding decisions.

## 2 Background

Social media has become a major environment for contemporary marketing communication, where brand meaning, consumer evaluation, and interaction with brand-related content are continuously shaped through platform-based exposure and participation [1]. Recent marketing research treats digital and social media marketing as a core research domain, while customer engagement is increasingly used to explain consumer responses that extend beyond immediate purchase outcomes to include interaction, participation, and relationship-building behaviors in digital settings [1,2]. In addition, influencer marketing has emerged as a major mechanism through which brands communicate product claims, symbolic meanings, and usage cues in social media environments. A recent meta-analytic review synthesizing 1531 effect sizes from 251 studies shows that influencer marketing has a meaningful effect on consumer behavior, while the strength of that effect varies according to source-related, message-related, and contextual factors [3].

Prior studies indicate that consumer responses to endorsed content are shaped by both endorser-related and content-related evaluations. Research on influencer advertising shows that credibility, expertise, and influencer-product congruence affect how consumers interpret sponsored content and respond to endorsements, while congruence between influencers, products, and consumers contributes to more favorable persuasion outcomes [4,5]. Recent work further suggests that authenticity and content-design features are important in shaping digital engagement with sponsored content. Evidence from sponsored video research shows that influencers’ authenticity management strategies affect digital engagement, while research on social media advertising features in retail settings indicates that entertainment and informativeness remain important drivers of downstream consumer responses [[6], [7], [8]].

Despite this accumulating evidence, openly available survey datasets that jointly capture endorser-related cues, content-related cues, and downstream brand-relevant outcomes within a single instrument remain scarce, particularly in emerging-market contexts. Existing studies often examine these endorser-related, content-related, and brand-relevant constructs separately, or rely on proprietary data that cannot be reused for replication, scale assessment, or comparative analysis [3,4,[6], [7], [8]]. Vietnam offers a relevant context for the dataset because social media advertising and influencer-led communication have become increasingly visible in its consumer markets, yet publicly accessible survey data on endorsed FMCG advertising in this setting remain virtually absent.

## 3 Data Description

This dataset contains screened survey responses from consumers in Vietnam who were eligible to evaluate one recalled social media-endorsed FMCG advertisement. The repository includes (i) two Microsoft Excel (.xlsx) dataset files, namely the raw dataset (1003 submitted responses) and the refined analytical dataset (563 valid responses), (ii) a separate codebook file, (iii) the informed consent form, and (iv) the administered questionnaire in English and Vietnamese. In both dataset files, each row represents one respondent and each column represents one survey variable. Variables include consent and SQ1–SQ5 screening items, 36 measurement indicators capturing the nine reflective constructs, and demographic and control variables. All responses are numerically coded; the codebook documents variable names, labels, response scales, and coding rules, including the valid range and meaning of grouped categorical values.

The questionnaire is organized into a consent and screening section, a main measurement block, and demographic and control questions, with all construct indicators rated on a five-point Likert scale. The nine reflective constructs are Credibility (CRED), Expertise (EXP), Congruence (CONG), Authenticity (AUTH), Entertainment (ENT), Informativeness (INF), Customer Experience (CX), Brand Association (BAS), and Engagement (ENG); the corresponding indicators are listed in Table 1.

**Table 1:** Measurement and operationalization.

*Construct*	*Code*	*Statement*	*Sources*
***Credibility***	*CRED1*	*The endorser in the social media-endorsed ad is believable.*	*[**3**,**9**]*
	*CRED2*	*I consider the endorser’s message to be trustworthy.*	
	*CRED3*	*The endorser appears honest when presenting the product.*	
	*CRED4*	*I feel confident relying on what the endorser says about the product.*	
***Expertise***	*EXP1*	*The endorser seems knowledgeable about the product.*	*[**3**,**9**]*
	*EXP2*	*The endorser appears experienced in this product category.*	
	*EXP3*	*The endorser seems qualified to introduce this product.*	
	*EXP4*	*The endorser gives the impression of having real understanding of the product.*	
***Congruence***	*CONG1*	*The endorser’s image fits well with the brand.*	*[**4**,**9**]*
	*CONG2*	*The endorser’s lifestyle matches the product being promoted.*	
	*CONG3*	*The endorsement feels natural.*	
	*CONG4*	*The endorser seems appropriate for promoting this product.*	
***Authenticity***	*AUTH1*	*The social media-endorsed ad content feels genuine.*	*[**6**,**7**]*
	*AUTH2*	*The endorsement appears sincere rather than forced.*	
	*AUTH3*	*The social media-endorsed ad gives the impression of reflecting a real opinion or experience.*	
	*AUTH4*	*The promoted message feels authentic rather than overly staged.*	
***Entertainment***	*ENT1*	*The social media-endorsed ad is enjoyable to watch.*	*[**2**,**8**]*
	*ENT2*	*The content makes me feel entertained.*	
	*ENT3*	*The social media-endorsed ad is interesting and engaging.*	
	*ENT4*	*The endorser’s presentation style makes the social media-endorsed ad more enjoyable.*	
***Informativeness***	*INF1*	*The social media-endorsed ad provides useful information about the product.*	*[**2**,**8**]*
	*INF2*	*The endorser clearly explains product benefits.*	
	*INF3*	*The content helps me better understand the product.*	
	*INF4*	*The social media-endorsed ad provides sufficient details for decision-making.*	
***Customer Experience***	*CX1*	*I feel comfortable watching this social media-endorsed ad.*	*[**9**]*
	*CX2*	*The social media-endorsed ad holds my attention while I am viewing it.*	
	*CX3*	*I feel satisfied after viewing this social media-endorsed ad.*	
	*CX4*	*Overall, my experience with this social media-endorsed ad is positive.*	
***Brand Association***	*BAS1*	*The social media-endorsed ad helps me form clear associations with the brand.*	*[**9**]*
	*BAS2*	*After seeing the social media-endorsed ad, the brand becomes easier to remember.*	
	*BAS3*	*The social media-endorsed ad makes the brand more meaningful to me.*	
	*BAS4*	*The social media-endorsed ad strengthens my positive image of the brand.*	
***Engagement***	*ENG1*	*I am willing to interact with this brand’s content on social media.*	*[**2**,**10**]*
	*ENG2*	*I would be willing to like, comment on, share, or save similar content from this brand.*	
	*ENG3*	*I am willing to search for more information about the brand after seeing this social media-endorsed ad.*	
	*ENG4*	*I would be interested in following future content related to this brand.*	

Table 2 reports the demographic and control characteristics of the 563 respondents retained in the refined analytical dataset. Female respondents account for 50.98% of the sample and male respondents for 45.47%, giving a relatively balanced gender distribution. Respondents aged 25–34 (32.50%) and 18–24 (25.58%) form the two largest age groups, reflecting a concentration of younger adults. Bachelor’s degree (38.19%) and college or diploma holders (29.13%) account for the largest educational shares, and office employees (34.28%) constitute the largest occupational group. Facebook (34.46%) and TikTok (23.27%) are the platforms on which endorsed advertising is most frequently encountered, influencers and KOLs (35.17%) are the most commonly noticed endorser type, and beverages (24.51%) and packaged food (20.07%) are the most frequently recalled FMCG categories.

**Table 2:** Sample characteristics (n = 563).

*Variable*	*Category*	*Frequency*	*Percentage (%)*
*Gender*	*Male*	*256*	*45.47*
	*Female*	*287*	*50.98*
	*Other*	*8*	*1.42*
	*Prefer not to say*	*12*	*2.13*
*Age group*	*18–24*	*144*	*25.58*
	*25–34*	*183*	*32.50*
	*35–44*	*114*	*20.25*
	*45–54*	*84*	*14.92*
	*55 or above*	*38*	*6.75*
*Education*	*High school or below*	*103*	*18.29*
	*College / Diploma*	*164*	*29.13*
	*Bachelor’s degree*	*215*	*38.19*
	*Postgraduate degree*	*81*	*14.39*
*Occupation*	*Student*	*94*	*16.70*
	*Office employee*	*193*	*34.28*
	*Business owner / Self-employed*	*107*	*19.01*
	*Freelancer*	*63*	*11.19*
	*Homemaker*	*29*	*5.15*
	*Other*	*77*	*13.68*
*Main platform*	*Facebook*	*194*	*34.46*
	*TikTok*	*131*	*23.27*
	*Instagram*	*112*	*19.89*
	*YouTube*	*88*	*15.63*
	*Other*	*38*	*6.75*
*Endorser type*	*Celebrity*	*127*	*22.56*
	*Influencer / KOL*	*198*	*35.17*
	*Content creator / reviewer*	*118*	*20.96*
	*Expert / professional reviewer*	*78*	*13.85*
	*Other*	*42*	*7.46*
*FMCG category*	*Packaged food*	*113*	*20.07*
	*Beverages*	*138*	*24.51*
	*Personal care*	*108*	*19.18*
	*Household products*	*82*	*14.56*
	*Health and hygiene products*	*79*	*14.03*
	*Other*	*43*	*7.64*
*Daily social media usage*	<*1 h*	*38*	*6.75*
	*1–2 h*	*108*	*19.18*
	*2–4 h*	*173*	*30.73*
	*4–6 h*	*139*	*24.69*
	>*6 h*	*105*	*18.65*

## 4 Experimental Design, Materials and Methods

The study employed a cross-sectional online survey design with non-probability purposive sampling. The questionnaire was administered through Google Forms and distributed via relevant Facebook groups in Vietnam between August and December 2025. The target population consisted of adult social media users in Vietnam who had recently encountered FMCG advertising endorsed by influencers, celebrities, KOLs, expert reviewers, or content creators and could recall one specific endorsed advertisement for evaluation.

Purposive sampling was adopted because the study required respondents who could credibly evaluate a recalled endorsed FMCG advertisement, a target population for which no comprehensive sampling frame is available. The sample is therefore appropriate for analytic and comparative purposes within this target population, but not for population-level inference. Participation required electronic informed consent and took approximately 10–12 min. No directly identifying personal information was collected.

The questionnaire began with an information sheet and an electronic consent statement. Respondents were informed about the research purpose, expected completion time, voluntary participation, anonymity, and the academic use of anonymized responses. The consent materials also stated that anonymized responses may be used for dissertations, journal articles, conference papers, research reports, and open research data archiving. Only respondents who agreed to participate were allowed to proceed to the screening section.

Five screening questions, recorded as SQ1–SQ5 and coded as binary variables (0 = not eligible, 1 = eligible), confirmed that respondents were at least 18 years old, actively used at least one major social media platform, had seen and interacted with an FMCG product promoted by an influencer, celebrity, KOL, or content creator on social media during the previous six months, and could clearly recall one specific endorsed advertisement for evaluation.

The raw dataset contained 1003 submitted cases. Of these, 57 did not provide consent and were excluded. Among the consenting cases, 329 failed at least one screening condition. A further 54 screening-qualified cases were removed during post-screening refinement because they exhibited low-quality response behavior, including mechanically repetitive patterns across long item blocks. The refined analytical dataset therefore retained 563 valid observations for analysis, and both files are released in the repository so that future users can trace the transition from the submitted pool to the final valid sample.

Measurement quality was assessed in SmartPLS 4 using partial least squares structural equation modeling (PLS-SEM), restricted to the reflective measurement model rather than full structural testing in line with the scope of a Data in Brief article. Internal consistency reliability was evaluated using Cronbach’s alpha and composite reliability, convergent validity was assessed using indicator loadings and average variance extracted, and discriminant validity was examined using the heterotrait–monotrait ratio. As presented in Table 3, all indicator loadings are above 0.84, ranging from 0.843 to 0.904. Cronbach’s alpha values range from 0.881 to 0.921, composite reliability values range from 0.918 to 0.944, and average variance extracted values range from 0.737 to 0.808. These results indicate satisfactory indicator reliability, internal consistency reliability, and convergent validity for all reflective constructs [11,12].

**Table 3:** Reliability and convergent validity of measurement scales.

*Construct*	*Indicator loadings*	*Cronbach’s alpha*	*Composite reliability (rho_c)*	*AVE*
*AUTH*	*0.855–0.881*	*0.888*	*0.923*	*0.749*
*BAS*	*0.890–0.904*	*0.921*	*0.944*	*0.808*
*CONG*	*0.852–0.885*	*0.892*	*0.925*	*0.755*
*CRED*	*0.843–0.885*	*0.888*	*0.923*	*0.749*
*CX*	*0.872–0.886*	*0.901*	*0.931*	*0.771*
*ENG*	*0.867–0.896*	*0.908*	*0.936*	*0.784*
*ENT*	*0.846–0.875*	*0.881*	*0.918*	*0.737*
*EXP*	*0.876–0.899*	*0.910*	*0.937*	*0.787*
*INF*	*0.844–0.879*	*0.887*	*0.922*	*0.747*

Table 4 reports the HTMT values for discriminant validity assessment. All HTMT values are below 0.85, with the highest value observed between BAS and ENG (0.734), indicating acceptable discriminant validity among the constructs [11,12].

**Table 4:** Discriminant validity using HTMT.

	*AUTH*	*BAS*	*CONG*	*CRED*	*CX*	*ENG*	*ENT*	*EXP*	*INF*
*AUTH*									
*BAS*	*0.352*								
*CONG*	*0.400*	*0.334*							
*CRED*	*0.387*	*0.225*	*0.344*						
*CX*	*0.524*	*0.634*	*0.525*	*0.487*					
*ENG*	*0.318*	*0.734*	*0.307*	*0.265*	*0.625*				
*ENT*	*0.352*	*0.284*	*0.317*	*0.345*	*0.490*	*0.270*			
*EXP*	*0.348*	*0.246*	*0.322*	*0.405*	*0.437*	*0.276*	*0.304*		
*INF*	*0.418*	*0.372*	*0.373*	*0.427*	*0.549*	*0.350*	*0.410*	*0.304*	

## Limitations

Several limitations of the dataset should be noted. First, the data were collected through a cross-sectional online survey distributed via Facebook groups, so the final sample may not fully represent all FMCG consumers or all social media users in Vietnam. Second, all variables are based on self-reported responses and on respondents’ ability to recall one specific endorsed FMCG advertisement, which may introduce recall bias as well as common-method bias associated with single-source self-reports. Third, the refined analytical dataset retains only respondents who passed all screening conditions and post-screening quality checks, so it reflects eligible and valid cases rather than the full pool of initial respondents. Finally, the data were collected between August and December 2025, and responses may vary across other periods, platforms, or campaign contexts.

## Ethics Statement

This study involved human participants in an anonymous online survey. Before accessing the questionnaire, respondents were presented with an information and consent page describing the study purpose, approximate completion time, voluntary participation, confidentiality protections, and the intended academic use of the data. Only those who provided electronic informed consent were allowed to proceed to the screening questions and the main questionnaire.

Participants were informed that no directly identifiable personal information, such as full name, identification number, phone number, or personal email address, would be collected. All responses were recorded anonymously and handled confidentially. The consent materials also stated that anonymized responses may be used for academic research purposes, including dissertations, journal articles, conference papers, research reports, and archiving in an open research data repository for transparency, verification, and replication.

Participation was entirely voluntary, and respondents could decline to participate or discontinue the survey at any time before submission without penalty. Because the study used an anonymous, non-invasive online questionnaire and did not collect sensitive personal identifiers, formal ethics committee approval was not required under applicable institutional regulations. The study was conducted in accordance with the ethical principles of the Declaration of Helsinki.

## CRediT Author Statement

**Thuy Duong Pham:** Conceptualization, Methodology, Investigation, Data collection, Data curation, Formal analysis, Writing - original draft. **Huong Thi Lan Le:** Supervision, Methodological validation, Writing - review and editing. **Hai Ninh Nguyen:** Supervision, Conceptualization support, Writing - review and editing, Correspondence.

## Data Availability

Mendeley DataSurvey dataset on social media-endorsed advertising in Vietnam’s FMCG sector (Original data)

## Appendix Supplementary materials

Image, application 1
