# Supplementary material for: Survey data on consumer responses to social media-endorsed FMCG advertising in Vietnam
Source: Data Brief. 2026 Jul 7;67:113060. doi: 10.1016/j.dib.2026.113060 (PMC13382170; doi:10.1016/j.dib.2026.113060)
Supplement: Supplementary file 1 [file mmc1.docx]

*Dear Sir/Madam,*

My name is Pham Thuy Duong, a PhD student in Business Administration at National Economics University. I am currently conducting a research project on the impacts of social media-endorsed advertising on brand association and consumer engagement in the fast-moving consumer goods (FMCG) sector in Vietnam.

Your participation in this survey is highly appreciated. The purpose of this study is to understand how consumers evaluate social media-endorsed advertising and how such evaluations influence their brand-related perceptions and engagement behavior.

The survey takes approximately 10-12 minutes to complete. Your participation is entirely voluntary, and you may refuse to participate or withdraw from the survey at any time without any negative consequences.

No directly identifiable personal information will be collected. All responses will be recorded anonymously and used for academic research purposes only. Data will be analyzed and reported in aggregated or anonymized form, and no individual participant will be identified. Anonymized data may also be used for scientific publications and shared in anonymized form through an open research data repository for scholarly purposes.

If you have any questions regarding this survey, please contact: [duongpt1207@gmail.com](mailto:duongpt1207@gmail.com)

Thank you very much for your time and support.

Sincerely,
**Pham Thuy Duong**

PhD student, Faculty of Business Administration.

National Economics University

**CONSENT STATEMENT FOR THE QUESTIONNAIRE**

**------**

After being informed about the purpose, content, rights, and responsibilities of research participants, please select one option below:

**⭘** *I have read and understood the information provided above and voluntarily agree to participate in this study. I understand that my responses will be recorded and kept anonymous, and may be used for academic research, scientific publications, and shared in anonymized form through an open research data repository for scholarly purposes.* **Continue to the screening questions**

**⭘** *I do not agree to participate in this study.* **The survey ends here. Thank you for your time.**

**SCREENING QUESTIONS**

**------**

1. Are you at least 18 years old?

**⭘** Yes

**⭘** No *(The survey ends here. Thank you for your time)*

2. Do you actively use at least one social media platform such as Facebook, TikTok, Instagram, or YouTube?

**⭘** Yes

**⭘** No *(The survey ends here. Thank you for your time)*

3. In the past 6 months, have you seen an FMCG product being promoted by an influencer, celebrity, KOL, or content creator on social media?

**⭘** Yes

**⭘** No *(The survey ends here. Thank you for your time)*

4. Have you ever interacted with such endorsed advertising content, for example by liking, commenting, sharing, saving, clicking, or searching for more information?

**⭘** Yes

**⭘** No *(The survey ends here. Thank you for your time)*

5. Can you recall one specific social media-endorsed advertisement for an FMCG product clearly enough to evaluate the following statements?

**⭘** Yes

**⭘** No *(The survey ends here. Thank you for your time)*

# **PART I. PERCEPTIONS OF SOCIAL MEDIA-ENDORSED** **ADVERTISING**

In this survey, social media-endorsed advertising refers to advertising content on social media platforms in which an influencer, celebrity, KOL, expert reviewer, or content creator promotes or recommends an FMCG product. Please answer the following questions based on one specific endorsed advertisement that you remember clearly. Use the scale below to indicate your level of agreement with each statement:

***1 = Strongly disagree, 2 = Disagree, 3 = Neutral, 4 = Agree, 5 = Strongly agree***

**1. Credibility**

|  | **1** | **2** | **3** | **4** | **5** |
| --- | --- | --- | --- | --- | --- |
| The endorser in the social media-endorsed ad is believable. |  |  |  |  |  |
| I consider the endorser’s message to be trustworthy. |  |  |  |  |  |
| The endorser appears honest when presenting the product. |  |  |  |  |  |
| I feel confident relying on what the endorser says about the product. |  |  |  |  |  |

**2. Expertise**

|  | **1** | **2** | **3** | **4** | **5** |
| --- | --- | --- | --- | --- | --- |
| The endorser seems knowledgeable about the product. |  |  |  |  |  |
| The endorser appears experienced in this product category. |  |  |  |  |  |
| The endorser seems qualified to introduce this product. |  |  |  |  |  |
| The endorser gives the impression of having real understanding of the product. |  |  |  |  |  |

## **3. Congruence**

|  | **1** | **2** | **3** | **4** | **5** |
| --- | --- | --- | --- | --- | --- |
| The endorser’s image fits well with the brand. |  |  |  |  |  |
| The endorser’s lifestyle matches the product being promoted. |  |  |  |  |  |
| The endorsement feels natural. |  |  |  |  |  |
| The endorser seems appropriate for promoting this product. |  |  |  |  |  |

**4. Authenticity**

|  | **1** | **2** | **3** | **4** | **5** |
| --- | --- | --- | --- | --- | --- |
| The social media-endorsed ad content feels genuine. |  |  |  |  |  |
| The endorsement appears sincere rather than forced. |  |  |  |  |  |
| The social media-endorsed ad gives the impression of reflecting a real opinion or experience. |  |  |  |  |  |
| The promoted message feels authentic rather than overly staged. |  |  |  |  |  |

**5. Entertainment**

|  | **1** | **2** | **3** | **4** | **5** |
| --- | --- | --- | --- | --- | --- |
| The social media-endorsed ad is enjoyable to watch. |  |  |  |  |  |
| The content makes me feel entertained. |  |  |  |  |  |
| The social media-endorsed ad is interesting and engaging. |  |  |  |  |  |
| The endorser’s presentation style makes the social media-endorsed ad more enjoyable |  |  |  |  |  |

**6. Informativeness**

|  | **1** | **2** | **3** | **4** | **5** |
| --- | --- | --- | --- | --- | --- |
| The social media-endorsed ad provides useful information about the product. |  |  |  |  |  |
| The endorser clearly explains product benefits. |  |  |  |  |  |
| The content helps me better understand the product. |  |  |  |  |  |
| The social media-endorsed ad provides sufficient details for decision-making. |  |  |  |  |  |

## **7. Customer Experience toward SMEA**

|  | **1** | **2** | **3** | **4** | **5** |
| --- | --- | --- | --- | --- | --- |
| I feel comfortable watching this social media-endorsed ad. |  |  |  |  |  |
| The social media-endorsed ad holds my attention while I am viewing it. |  |  |  |  |  |
| I feel satisfied after viewing this social media-endorsed ad. |  |  |  |  |  |
| Overall, my experience with this social media-endorsed ad is positive. |  |  |  |  |  |

# **PART II. BRAND-RELATED OUTCOMES**

## **8. Brand Association**

|  | **1** | **2** | **3** | **4** | **5** |
| --- | --- | --- | --- | --- | --- |
| The social media-endorsed ad helps me form clear associations with the brand. |  |  |  |  |  |
| After seeing the social media-endorsed ad, the brand becomes easier to remember. |  |  |  |  |  |
| The social media-endorsed ad makes the brand more meaningful to me. |  |  |  |  |  |
| The social media-endorsed ad strengthens my positive image of the brand. |  |  |  |  |  |

## **9. Engagement**

|  | **1** | **2** | **3** | **4** | **5** |
| --- | --- | --- | --- | --- | --- |
| I am willing to interact with this brand’s content on social media. |  |  |  |  |  |
| I would be willing to like, comment on, share, or save similar content from this brand. |  |  |  |  |  |
| I am willing to search for more information about the brand after seeing this social media-endorsed ad. |  |  |  |  |  |
| I would be interested in following future content related to this brand. |  |  |  |  |  |

# **PART III. PERSONAL INFORMATION**

**1. Age: ⭘** 18–24 **⭘** 25–34 **⭘** 35–44 **⭘** 45–54 **⭘** 55 or above

**2. Gender: ⭘** Male **⭘** Female **⭘** Other **⭘** Prefer not to say

**3. Education level: ⭘** High school or below **⭘** College / Diploma

**⭘** Bachelor’s degree **⭘** Postgraduate degree

**4. Occupation: ⭘** Student **⭘** Office employee **⭘** Business owner / Self-employed

**⭘** Freelancer **⭘** Homemaker **⭘** Other

**5. Which social media platform do you use most frequently to view endorsed advertising content?**

**⭘** Facebook **⭘** TikTok **⭘** Instagram **⭘** YouTube **⭘** Other

**6. Which type of endorser do you pay most attention to?**

**⭘** Celebrity **⭘** Influencer / KOL **⭘** Content creator / reviewer

**⭘** Expert / professional reviewer **⭘** Other

**7. Which FMCG product category did the advertisement you recalled mainly belong to?**

**⭘** Packaged food **⭘** Beverages **⭘** Personal care

**⭘** Household products **⭘** Health and hygiene products **⭘** Other

**8. How often do you use social media in a typical day?**

**⭘** Less than 1 hour **⭘** 1–2 hours **⭘** 2–4 hours

**⭘** 4–6 hours **⭘** More than 6 hours
